# Serum Metabolomics Investigation of Humanized Mouse Model of Dengue Virus Infection

**DOI:** 10.1128/JVI.00386-17

**Published:** 2017-06-26

**Authors:** Liang Cui, Jue Hou, Jinling Fang, Yie Hou Lee, Vivian Vasconcelos Costa, Lan Hiong Wong, Qingfeng Chen, Eng Eong Ooi, Steven R. Tannenbaum, Jianzhu Chen, Choon Nam Ong

**Affiliations:** a Interdisciplinary Research Group in Infectious Diseases, Singapore-MIT Alliance for Research and Technology (SMART), Singapore; b Saw Swee Hock School of Public Health, National University of Singapore, Singapore; c KK Research Centre, KK Women's and Children's Hospital, Singapore; d Humanized Mouse Unit, Institute of Molecular and Cell Biology, Agency for Science, Technology and Research (A*STAR), Singapore; e Department of Microbiology, Yong Loo Lin School of Medicine, National University of Singapore, Singapore; f Emerging Infectious Diseases Program, Duke-NUS Graduate Medical School, Singapore; g Departments of Biological Engineering and Chemistry, Massachusetts Institute of Technology, Cambridge, Massachusetts, USA; h Department of Biology, Massachusetts Institute of Technology, Cambridge, Massachusetts, USA; i NUS Environment Research Institute, Singapore; University of Southern California

**Keywords:** dengue fever, humanized mice, mass spectrometry, metabolomics, systems biology

## Abstract

Dengue is an acute febrile illness caused by dengue virus (DENV) and a major cause of morbidity and mortality in tropical and subtropical regions of the world. The lack of an appropriate small-animal model of dengue infection has greatly hindered the study of dengue pathogenesis and the development of therapeutics. In this study, we conducted mass spectrometry-based serum metabolic profiling from a model using humanized mice (humice) with DENV serotype 2 infection at 0, 3, 7, 14, and 28 days postinfection (dpi). Forty-eight differential metabolites were identified, including fatty acids, purines and pyrimidines, acylcarnitines, acylglycines, phospholipids, sphingolipids, amino acids and derivatives, free fatty acids, and bile acid. These metabolites showed a reversible-change trend—most were significantly perturbed at 3 or 7 dpi and returned to control levels at 14 or 28 dpi, indicating that the metabolites might serve as prognostic markers of the disease in humice. The major perturbed metabolic pathways included purine and pyrimidine metabolism, fatty acid β-oxidation, phospholipid catabolism, arachidonic acid and linoleic acid metabolism, sphingolipid metabolism, tryptophan metabolism, phenylalanine metabolism, lysine biosynthesis and degradation, and bile acid biosynthesis. Most of these disturbed pathways are similar to our previous metabolomics findings in a longitudinal cohort of adult human dengue patients across different infection stages. Our analyses revealed the commonalities of host responses to DENV infection between humice and humans and suggested that humice could be a useful small-animal model for the study of dengue pathogenesis and the development of dengue therapeutics.

**IMPORTANCE** Dengue virus is the most widespread arbovirus, causing an estimated 390 million dengue infections worldwide every year. There is currently no effective treatment for the disease, and the lack of an appropriate small-animal model of dengue infection has greatly increased the challenges in the study of dengue pathogenesis and the development of therapeutics. Metabolomics provides global views of small-molecule metabolites and is a useful tool for finding metabolic pathways related to disease processes. Here, we conducted a serum metabolomics study on a model using humanized mice with dengue infection that had significant levels of human platelets, monocytes/macrophages, and hepatocytes. Forty-eight differential metabolites were identified, and the underlying perturbed metabolic pathways are quite similar to the pathways found to be altered in dengue patients in previous metabolomics studies, indicating that humanized mice could be a highly relevant small-animal model for the study of dengue pathogenesis and the development of dengue therapeutics.

## INTRODUCTION

Dengue is an acute febrile illness caused by dengue virus (DENV) and is a major cause of morbidity and mortality in tropical and subtropical regions of the world. It is arguably the most important arboviral disease globally, with an estimated 390 million infections occurring every year, nearly 100 million of which are clinically apparent (1, 2). In the absence of an effective antiviral drug and with the rapid spread of DENV, which is now circulating in Asia, Africa, and the Americas, dengue has become a major public health threat worldwide (3).

Dengue manifests in a wide spectrum of clinical symptoms ranging from mild dengue fever (DF) to life-threatening dengue hemorrhagic fever/dengue shock syndrome (DHF/DSS). The symptoms of dengue are usually accompanied by hematological changes, such as leukopenia and thrombocytopenia in mild cases and plasma leakage, hemorrhage, or organ impairment, such as liver damage, in severe cases (4). A major challenge in the study of dengue pathogenesis and the development of therapeutics for dengue is the lack of an appropriate small-animal model of dengue infection, despite efforts to develop relevant mouse models (5–7). Among these models, the development of humice, which are immunodeficient mice stably reconstituted with human immune cells, is appealing because it makes it possible to study DENV infection in a human-like context. We have successfully constructed humice by adoptive transfer of human CD34^+^ fetal liver cells into NOD-*scid Il2rg*^−/−^ (NSG) mice, which develop significant levels of human platelets, monocytes/macrophages, and hepatocytes (8). Infection of these mice with DENV serotype 2 (DENV2) recapitulates some of the characteristic features of DENV infection in humans, including transient leukopenia, thrombocytopenia, and liver damage (8).

Metabolomics is a rapidly emerging fields of “omics” research that provides an analysis of the global metabolite changes in biological systems in response to biological stimuli or perturbations (9). As the end products of cellular regulatory processes, metabolites not only play critical roles in biology, but also provide a functional readout of cellular biochemistry, which is important for finding metabolic pathways related to disease processes (10, 11). In our previous study, a systematic characterization of serum metabolome changes in a longitudinal cohort of adult dengue patients across three infection stages (febrile, defervescence, and convalescence) was conducted, and many metabolic pathways linked to disease progression were identified (12). In the present study, we extended our metabolomics study to serum samples from humice at multiple time points after DENV infection, with the aim of comparing the similarities and differences of metabolome changes between dengue patients and humice. Our results showed that most perturbed metabolic pathways were common between dengue patients and DENV-infected humice, indicating humice could be a highly relevant small-animal model for the study of dengue pathogenesis and the development of therapeutics.

## RESULTS

### Systemic DENV infection and characteristic responses in humice.

**FIG 1:**
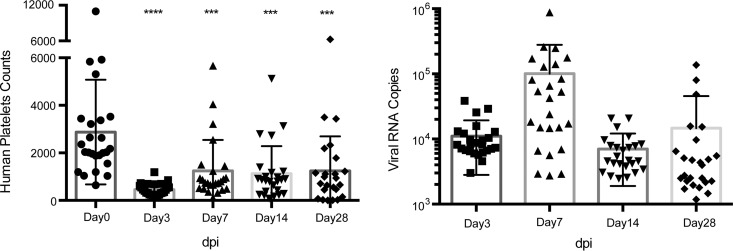
The thrombocytopenia of human platelets and the viral RNA load were detected in dengue virus-infected humanized mice. Twenty-five humanized mice were infected with 1 × 10^7^ PFU Den2/07K2861 virus intravenously (i.v.). Subsequently, the mice were bled at the indicated times (dpi), and the whole blood was used for platelet counting, while the serum was used for viral load measurement. (A) The human platelets dropped significantly after virus infection from 3 dpi to 28 dpi compared with preinfection levels. Ten microliters of humanized mouse whole blood was stained for human CD41 and mouse CD41, and then human and mouse platelets were counted by flow cytometry using fluorescent beads. (B) Viremia was measured by virus-specific qRT-PCR in serum. The serum samples were collected at the indicated times (dpi) and used to extract viral RNA. The data shown are the numbers of viral RNA copies per microliter of serum. The qRT-PCR detection limit was approximately 1,000 copies.

After the construction of humice, mouse peripheral blood cells were analyzed for human leukocyte reconstruction, which showed the presence of human monocytes/macrophages and platelets in the humice. Humice with 10% or more reconstitution levels [using the following formula: human CD45^+^ cells/(human CD45^+^ cells + mouse CD45^+^ cells) × 100] in the blood were used for subsequent experiments. After intravenous infection with 1 × 10^7^ PFU DENV2 07K2861, viremia was detected in the infected humice from 3 days postinfection (dpi) and persisted until 28 dpi by quantitative reverse transcription (qRT)-PCR for the Env gene of viral RNA. The viremia peaked at 7 dpi, which was consistent with our previous studies (Fig. 1A) (8). Human-specific thrombocytopenia was also observed in the humice (Fig. 1B), which specifically identified human and mouse platelets by human CD41 (hCD41) and mouse CD41 (mCD41) cell surface markers. The human platelets dropped significantly from 2,875 (mean value) before infection to 458 (mean value) by day 3 dpi. Overall, based on these model characteristics, serum samples were collected at 0, 3, 7, 14, and 28 dpi, corresponding to the febrile, defervescence, and convalescence phases in humans, for the metabolomics study.

### Metabolomics analysis by LC-MS.

We characterized serum metabolome changes of both humice and NSG mice with DENV infection using liquid chromatography-mass spectrometry (LC-MS). Both reverse-phase ultrahigh performance liquid chromatography (RP-UHPLC) and hydrophilic interaction ultrahigh performance liquid chromatography (HILIC-UHPLC) techniques were adopted here to expand the coverage of both polar and nonpolar metabolites (13). In order to obtain reliable metabolic profiles of the samples, it was important to ensure the robustness of the analytical method. We first evaluated the stability and reproducibility of the LC-MS method by performing principal-components analysis (PCA) on all the samples, including the 7 quality control (QC) samples (14). As shown in Fig. S1 in the supplemental material, the QC samples are clustered in PCA score plots of sera, indicating good stability and reproducibility of the chromatographic separation during the whole sequence.

The transient viremia, leukopenia, and thrombocytopenia of the model with DENV infection suggested the illness was self-limiting in humice. This is similar to DF patients, who recover uneventfully after a period of acute illness. Thus, we adopted the same strategy used in our previous metabolomics study on DF patients by focusing on the identification of the differential metabolites that showed a reversible-change trend along the time course, because these metabolites were more relevant to the disease progression (12).

### Metabolome changes between humice and NSG mice without DENV infection.

**FIG 2:**
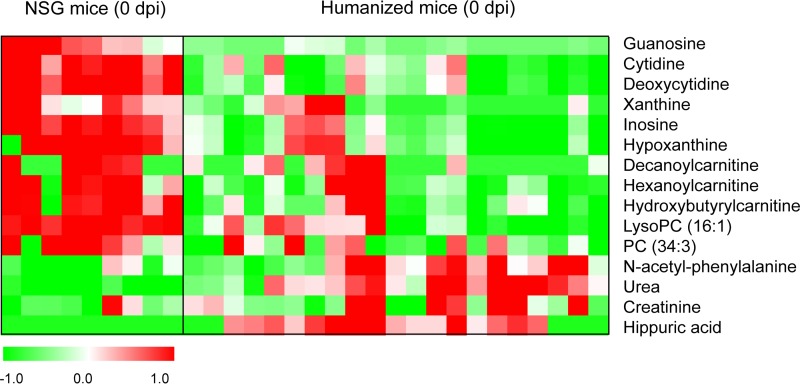
Heat map of identified differential metabolites between humice and NSG mice without dengue infection. Each row shows the ion intensity for a specific metabolite after mean centering and unit variance scaling of the data. Each column shows the serum metabolic profiles of humice and NSG mice at 0 dpi.

We first studied metabolome differences between humice and NSG mice before DENV infection to determine how the adoptive transfer of human CD34^+^ fetal liver cells into NSG mice would change their metabolome. About 120 differential features between humice and NSG mice were found, and the structures of 15 metabolites were identified, including purines and pyrimidines, acylcarnitines, and phospholipids (Fig. 2). Compared to NSG mice, most of these differential metabolites showed lower levels in humice, and the levels of purines and pyrimidines decreased most significantly (3 to 4 times). Meanwhile, the levels of reconstitution in humice showed significant negative correlations to the two nucleosides cytidine (*r* = −0.64; *P* = 0.002) and deoxycytidine (*r* = −0.61; *P* = 0.003) by Pearson correlation analysis (see Fig. S2 in the supplemental material).

### Metabolome changes of NSG mice and humice with DENV infection.

Although NSG mice with DENV infection did not show viremia or any of the characteristic features of dengue patients, we still observed about 30 differential features that showed a reversible-change trend between noninfected and DENV-infected NSG mice. Five of these metabolites, hypoxanthine, deoxycytidine, and 3 phospholipids, were identified (see Fig. S3 in the supplemental material). The phospholipids first decreased, reached their lowest levels at around 7 dpi, and then gradually returned to the control levels at 28 dpi. Conversely, hypoxanthine and deoxycytidine were increased, reached their highest levels at 3 or 7 dpi, and then returned to the control levels at 28 dpi.

**FIG 3:**
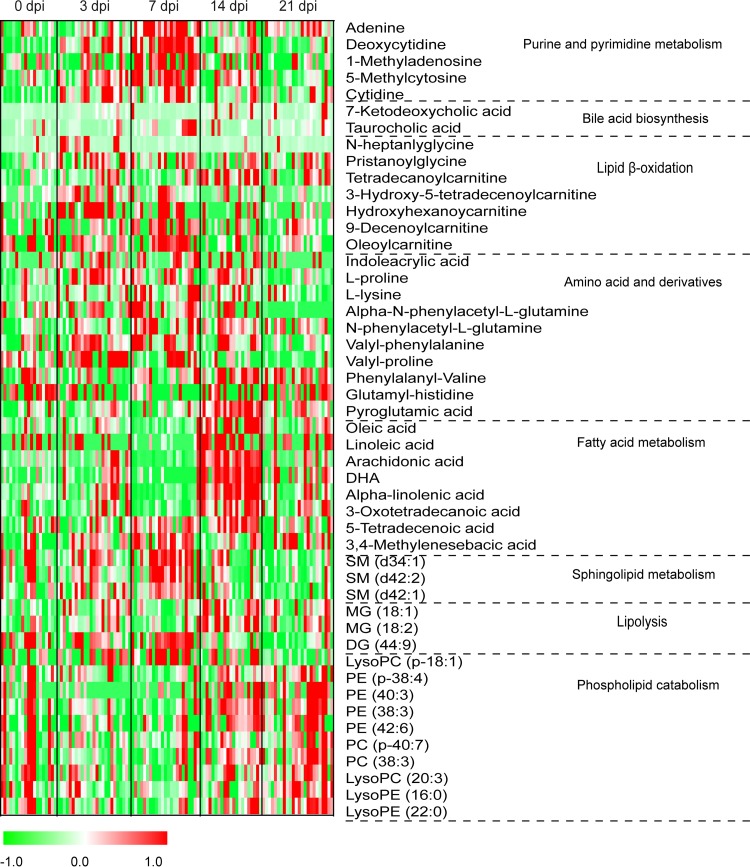
Heat map of identified differential metabolites in humice with dengue infection. Each row shows the ion intensity for a specific metabolite after mean centering and unit variance scaling of the data. The columns show the serum metabolic profiles of humice with DENV infection at 0, 3, 7, 14, and 28 dpi.

**FIG 4:**
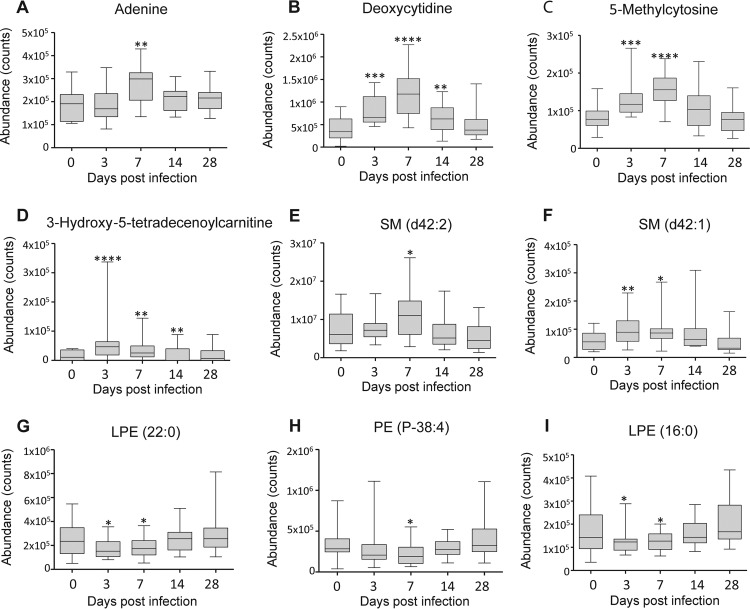
Typical change trends based on differential metabolite classes in humice with dengue infection. (A) Elevated change trend of adenine (purine). (B) Elevated change trend of deoxycytinine (pyrimidine). (C) Elevated change trend of 5-methylcytosine (pyrimidine). (D) Elevated change trend of 3-hydroxy-5-tetradecenoylcarnitine (acylcarnitine). (E) Elevated change trend of SM d42:2 (sphingolipid). (F) Elevated change trend of SM d42:1 (sphingolipid). (G) Decreased change trend of lyoPE (LPE) 22:0 (phospholipid). (H) Decreased change trend of PE P-38:4 (phospholipid). (I) Decreased change trend of LPE 16:0 (phospholipid). The bottom and the top of each box are the 25th and the 75th percentiles, and the black line near the middle of the box is the median peak area of the metabolite. *, *P* < 0.05; **, *P* < 0.01; ***, *P* < 0.001; ****, *P* < 0.0001 by the Kruskal-Wallis test. The statistical comparison was with control levels. Whiskers are from minimum to maximum.

In humice with DENV infection, about 400 differential features showed a reversible-change trend, and 48 metabolites were identified, including purines and pyrimidines, acylcarnitines, acylglycines, phosphatidylcholines (PCs), phosphatidylethanolamines (PEs), lysophosphatidylcholines (lysoPCs), lysophosphatidylethanolamines (lysoPEs), amino acids and derivatives, free fatty acids (FFAs), sphingomyelins (SMs), monoacylglycerides (MGs), diacylglyceride (DG), and bile acids (Fig. 3 and Table 1). Most of these metabolites were significantly perturbed at 3 or 7 dpi and normalized to control levels at 14 or 28 dpi, suggesting that they might serve as prognostic markers of the disease in humice. The metabolites within each metabolite class generally showed similar change trends. For example, the 7 FFAs, 5 purines and pyrimidines, 4 acylcarnitines, and 3 SMs showed an elevated trend at 3 and 7 dpi and returned to the control levels at 14 and 28 dpi (Fig. 4A to F). Conversely, 9 out of the 10 phospholipids showed a decreased trend at 3 and 7 dpi and returned to the control levels at 14 and 28 dpi (Fig. 4G to I). The classes of these different metabolites and their reversible-change trends were similar to our previous findings in a longitudinal cohort of adult dengue patients across three infection stages (12). Specifically, FFA, acylcarnitine, bile acid, purine, SM, MG, and DG showed an increased trend in both humice and dengue patients at the early or acute stage of DENV infection and gradually returned to the control levels at the late stage. Conversely, phospholipids mainly displayed a downward trend in both humice and dengue patients at the early stage and returned to the control levels at the late stage.

**TABLE 1:** Identified differential metabolites in humanized mice with dengue infection

HMDB	Mass (Da)		Metabolite	Chemical formula	Change trend	Pathway	Dengue patients
	Accurate	Theoretical					
HMDB00207	282.24945	282.2558803	Oleic acid	C_18_H_34_O_2_	↑	Fatty acid biosynthesis	Yes
HMDB00673	280.2353	280.2402302	Linoleic acid	C_18_H_32_O_2_	↑	Fatty acid biosynthesis	Yes
HMDB01043	304.2328	304.2402302	Arachidonic acid	C_20_H_32_O_2_	↑	Fatty acid biosynthesis	Yes
HMDB02183	328.23312	328.2402302	DHA	C_22_H_32_O_2_	↑	Fatty acid biosynthesis	Yes
HMDB01388	278.219	278.2245802	Alpha-linolenic acid	C_18_H_30_O_2_	↑	Fatty acid biosynthesis	Yes
HMDB10730	242.18243	242.1881946	3-Oxotetradecanoic acid	C_14_H_26_O_3_	↑	Fatty acid biosynthesis	No
HMDB59729	226.1204	226.1205091	3,4-Methylenesebacic acid	C_12_H_18_O_4_	↑	Fatty acid biosynthesis	No
HMDB00521	226.19693	226.1932801	5-Tetradecenoic acid	C_14_H_26_O_2_	↑	Fatty acid oxidation	No
HMDB11567	356.286	356.2926597	MG(18:1)	C_21_H_40_O_4_	↑	Lipolysis	Yes
HMDB11568	354.26956	354.2770097	MG(18:2)	C_21_H_38_O_4_	↑	Lipolysis	Yes
HMDB07700	718.556	718.5536255	DG(22:4/22:5/0:0)	C_47_H_74_O_5_	↑	Lipolysis	Yes
HMDB00034	135.05618	135.0544952	Adenine	C_5_H_5_N_5_	↑	Purine metabolism	No
HMDB00014	227.09274	227.0906059	Deoxycytidine	C_9_H_13_N_3_O_4_	↑	Pyrimidine metabolism	No
HMDB03331	281.11356	281.112404	1-Methyladenosine	C_11_H_15_N_5_O_4_	↑	Pyrimidine metabolism	No
HMDB02894	125.05924	125.0589119	5-Methylcytosine	C_5_H_7_N_3_O	↑	Pyrimidine metabolism	No
HMDB00089	243.08305	243.0855205	Cytidine	C_9_H_13_N_3_O_5_	↑	Pyrimidine metabolism	No
HMDB00391	406.2621	406.2719243	7-Ketodeoxycholic acid	C_24_H_38_O_5_	↑	Bile acid biosynthesis	No
HMDB00036	515.28796	515.2916735	Taurocholic acid	C_26_H_45_NO_7_S	↑	Bile acid biosynthesis	No
HMDB13010	187.1234	187.1208434	*N*-Heptanoylglycine	C_9_H_17_NO_3_	↑	Acylglycine	No
HMDB13303	355.30783	355.3086442	Pristanoylglycine	C_21_H_41_NO_3_	↑	Acylglycine	No
HMDB05066	371.29556	371.3035588	Tetradecanoylcarnitine	C_21_H_41_NO_4_	↑	Lipid β-oxidation	Yes
HMDB13330	385.27936	385.2828234	3-Hydroxy-5-tetradecenoylcarnitine	C_21_H_39_NO_5_	↑	Lipid β-oxidation	No
HMDB13131	275.17087	275.1732729	Hydroxyhexanoycarnitine	C_13_H_25_NO_5_	↑	Lipid β-oxidation	No
HMDB13205	313.225	313.2253085	9-Decenoylcarnitine	C_17_H_31_NO_4_	↑	Lipid β-oxidation	Yes
HMDB05065	425.34814	425.350509	Oleoylcarnitine	C_25_H_47_NO_4_	↑	Lipid β-oxidation	Yes
HMDB00267	129.04066	129.042593	Pyroglutamic acid	C_5_H_7_NO_3_	↑	Glutathione metabolism	Yes
HMDB00734	187.0611	187.0633285	Indoleacrylic acid	C_11_H_9_NO_2_	↑	Tryptophan metabolism	Yes
HMDB00162	115.0609	115.0633285	l-Proline	C_5_H_9_NO_2_	↑	Arginine and proline metabolism	No
HMDB00182	146.1077	146.1055277	l-Lysine	C_6_H_14_N_2_O_2_	↑	Lysine degradation	No
HMDB06344	264.10547	264.111007	Alpha-*N*-phenylacetyl–l-glutamine	C_13_H_16_N_2_O_4_	↑	Phenylalanine metabolism	No
HMDB00512	207.09024	207.0895433	*N*-Acetyl-l-phenylalanine	C_11_H_13_NO_3_	↑	Phenylalanine metabolism	No
HMDB29134	264.1464	264.1473925	Valyl-phenylalanine	C_14_H_20_N_2_O_3_	↑	Dipeptide	No
HMDB29135	214.1325	214.1317425	Valyl-proline	C_10_H_18_N_2_O_3_	↑	Dipeptide	No
HMDB29134	264.15723	264.1473925	Valyl-phenylalanine	C_14_H_20_N_2_O_3_	↓	Dipeptide	No
HMDB28821	283.103	283.1042446	Glutamyl-histidine	C_11_H_15_N_4_O_5_	↓	Dipeptide	No
HMDB13464	702.5609	702.5675748	SM(d18:0/16:1)	C_39_H_79_N_2_O_6_P	↑	Sphingolipid metabolism	Yes
HMDB12107	812.6705	812.6771252	SM(d18:1/24:1)	C_47_H_93_N_2_O_6_P	↑	Sphingolipid metabolism	Yes
HMDB12095	814.6839	814.6927753	SM(d18:0/24:1)	C_47_H_95_N_2_O_6_P	↑	Sphingolipid metabolism	No
HMDB13122	507.3682	507.368875	LysoPC(P-18:0)	C_26_H_54_NO_6_P	↑	Phospholipid catabolism	Yes
HMDB05779	751.5493	751.5515904	PE(P-38:4)	C_43_H_78_NO_7_P	↓	Phospholipid catabolism	Yes
HMDB09041	797.5837	797.5934552	PE(40:3)	C_45_H_84_NO_8_P	↓	Phospholipid catabolism	No
HMDB09551	769.5633	769.5621551	PE(38:3)	C_43_H_80_NO_8_P	↓	Phospholipid catabolism	Yes
HMDB09243	819.58105	819.5778051	PE(42:6)	C_47_H_82_NO_8_P	↓	Phospholipid catabolism	Yes
HMDB08754	815.5827	815.5828905	PC(P-40:7)	C_48_H_82_NO_7_P	↓	Phospholipid catabolism	No
HMDB08020	811.60394	811.6091052	PC(38:3)	C_46_H_86_NO_8_P	↓	Phospholipid catabolism	Yes
HMDB10393	545.34375	545.3481395	LysoPC(20:3)	C_28_H_52_NO_7_P	↓	Phospholipid catabolism	No
HMDB11473	453.28265	453.2855393	LysoPE(16:0)	C_21_H_44_NO_7_P	↓	Phospholipid catabolism	Yes
HMDB11490	537.3757	537.3794397	LysoPE(22:0)	C_27_H_56_NO_7_P	↓	Phospholipid catabolism	No

We used the pathway analysis tool MetaboAnalyst to determine the underlying biochemical pathways revealed by these different metabolites. Major perturbed metabolic pathways in humice after DENV infection included purine and pyrimidine metabolism, fatty acid β-oxidation, phospholipid catabolism, arachidonic acid and linoleic acid metabolism, sphingolipid metabolism, tryptophan metabolism, phenylalanine metabolism, lysine biosynthesis and degradation, and bile acid biosynthesis (see Fig. S4 in the supplemental material). Most of the altered metabolic pathways were similar to the perturbed pathways in dengue patients in our previous reports (12).

## DISCUSSION

Dengue is a major global public health threat, and the lack of an appropriate small-animal model of dengue infection has greatly hindered the study of dengue pathogenesis and the development of therapeutics. We have successfully constructed humice with significant levels of human platelets, monocytes/macrophages, and hepatocytes. Infection of the humice with DENV2 could recapture some of the characteristic features of DENV infection in humans, including transient leukopenia, thrombocytopenia, and liver damage. We have previously studied serum metabolome changes using LC-MS in a longitudinal cohort of adult dengue patients across three prominent infection stages (early febrile, defervescence, and convalescence stages) and identified a variety of metabolic pathways linked to dengue progression. In the present study, a similar metabolomics study was conducted on serum samples from humice at multiple time points after DENV infection with the aim of comparing the similarities and differences of metabolome changes between dengue patients and humice infected with DENV. Our results showed that most disturbed metabolic pathways were common between dengue patients and DENV-infected humice, suggesting humice could be a highly relevant small-animal model for the study of dengue pathogenesis and the development of dengue therapeutics.

DENV-infected NSG mice did not show viremia or any characteristic features of human dengue patients, but we still observed some differential features between infected and noninfected NSG mice, indicating that our analytical approach is sensitive enough to capture the metabolome changes of the NSG mice even without apparent phenotypical changes. Conversely, viremia and certain features of human dengue patients, like transient leukopenia, thrombocytopenia, and liver damage, were observed in infected humice. Correspondingly, a much larger number of differential features were observed in infected humice than in noninfected humice. Thirty-eight differential metabolites were identified, which belonged to different metabolite classes, such as purine and pyrimidine, acylcarnitine, acylglycine, phospholipid, sphingolipid, amino acids and derivatives, free fatty acid, and bile acid. In our previous metabolomics study, we found that metabolites from all these classes were also significantly altered in adult patients with DENV infection compared to healthy controls (12). However, more purines and pyrimidines and amino acids and derivatives but fewer free fatty acids were found in the current study. This could be due to the change of analytical platform from reversed phase to HILIC, which could capture more polar compounds. Nevertheless, these results showed many similarities in host responses to DENV infection between humans and humice.

Dengue in humans is a dynamic disease, and after the incubation period, the illness begins abruptly and is followed by the three phases—febrile (days 0 to 4 after onset of fever), critical (days 5 to 7 after onset of fever), and recovery. The mild form of dengue is generally self-limiting, and patients recover uneventfully after 5 to 7 days of acute illness. Our previous metabolomics study on patients with mild dengue showed that the differential metabolites between dengue patients and healthy controls were significantly perturbed during the early febrile and critical stages and normalized to control levels at the recovery stage, which was reflective of dengue disease progression. Similarly, the transient viremia, leukopenia, and thrombocytopenia in humice with DENV infection suggested the illness was self-limiting in the model, as well. Indeed, we identified 38 differential metabolites between infected and noninfected humice, which showed a reversible-change trend across the disease course—they were significantly perturbed at 3 or 7 dpi and returned to control levels at 14 or 28 dpi. Furthermore, the same metabolite class showed similar change trends between humice and dengue patients. For example, the purines and acylcarnitines showed an elevated trend at early febrile and critical stages in dengue patients, and similarly, they had an elevated trend at 3 and 7 dpi in infected humice. On the other hand, the majority of the phospholipids showed a decreased trend at febrile and critical stages in dengue patients and at 3 and 7 dpi in infected humice. Altogether, the same classes of metabolites were perturbed with similar change trends over the disease course in infected humice and dengue patients, and the differential metabolites found in this study could serve as prognostic markers to monitor dengue progression in humice.

The differential metabolites in the humouse model belonged to a variety of metabolic pathways, most of which were also significantly altered in dengue patients. The commonly altered pathways in both infected humice and dengue patients include fatty acid β-oxidation, phospholipid catabolism, sphingolipid metabolism, purine and pyrimidine metabolism, arachidonic acid and linoleic acid metabolism, tryptophan metabolism, phenylalanine metabolism, and bile acid biosynthesis. Among these pathways, bile acid biosynthesis is closely linked to liver impairment. DENV can infect the hepatocytes and have adverse effects on liver functions in dengue patients, which is monitored by liver enlargement and elevated transaminases among clinical cases (15–17). The levels of liver transaminases were indeed significantly increased in the sera of infected humice. Bile acids are toxic to cells at abnormally high levels (18), and increased bile acid biosynthesis might contribute to liver pathogenesis in infected humice. On the other hand, the steroid hormone biosynthesis and heme degradation pathways were perturbed in dengue patients, but not in humice with DENV infection. DENV infects a multitude of cells and organs in humans (19, 20), and although significant levels of human monocytes/macrophages and hepatocytes are present in humice, many types of cells are still lacking. Thus, although many metabolic pathways were commonly perturbed in infected humice and dengue patients, some altered pathways in dengue patients could not be found in humice.

The main limitation of the study is the relatively large variation of individual humanized mice in response to the infection, and larger numbers of humice, 25 in this study, may be needed. Nevertheless, our analyses provide a detailed description of the metabolome changes in humice with DENV infection, and the data revealed many commonalities of host responses to DENV infection between humans and humice, suggesting that this small-animal model could be used for the study of dengue pathogenesis and the development of dengue therapeutics.

## MATERIALS AND METHODS

### Ethics statement.

Human fetal livers at 15 to 23 weeks of gestation were collected in accordance with the institutional ethical guidelines of the National University Hospital of Singapore and National University of Singapore (NUS). All the women gave written informed consent for the donation of their fetal tissue for research. All experiments involving mice were carried out in strict accordance with the National Advisory Committee for Laboratory Animal Research (NACLAR) guidelines (Guidelines on the Care and Use of Animals for Scientific Purposes) in facilities licensed by the Agri-Food and Veterinary Authority of Singapore (AVA), the regulatory body of the Singapore Animals and Birds Act. The humouse protocol (R16-0158) was approved by the Institutional Animal Care and Use Committee (IACUC), National University of Singapore. The mice were monitored every day after infection, and any mouse with 30% body weight loss was euthanatized immediately.

### Construction of humice and infection with dengue virus.

The humouse model was developed as in our previous report (21). Briefly, human fetal livers at 15 to 23 weeks of gestation were collected, and the human CD34^+^ cells were isolated and purified using a CD34-positive magnetic selection kit (Stem Cell Technologies). CD34^+^ cells (2 × 10^5^) were injected into sublethally irradiated NSG pups within 24 to 48 h of birth. The mice were analyzed for the human leukocyte reconstitution rate by staining mouse CD45 and human CD45 at 12 weeks after CD34^+^ cell injection. Twenty-five humanized mice and 10 NSG mice were infected by injecting 1 × 10^7^ PFU of the concentrated DENV2 (strain 07K2861) in 200 μl of RPMI 1640 medium through the tail vein. Sera were collected from both NSG mice and humice at 0, 3, 7, 14, and 28 dpi for flow cytometry assays, measuring viral RNA levels, platelet counts, and metabolomics analysis. The viral RNA extraction and qRT-PCR assay and platelet counts by fluorescence-activated cell sorting (FACS) were described in our previous paper (8).

### Serum sample preparation.

The procedure for serum sample preparation was the same as in our previously published report (12, 22). Briefly, a 50-μl volume of serum was thawed at 4°C, and proteins were precipitated with 200 μl ice-cold methanol, which contained 5 μg/ml 9-fluorenylmethoxycarbonyl-glycine as an internal standard. After vortexing, the mixture was centrifuged at 16,000 rpm for 10 min at 4°C, and the supernatant was collected and evaporated to dryness in a vacuum concentrator. The dry extracts were then resuspended in 100 μl of 98:2 water/methanol for LC-MS analysis. QC samples were prepared by mixing equal amounts of sera from all the samples and processed as real samples. All samples were kept at 4°C and analyzed in a random manner. The QC sample was run after every 15 samples to monitor the stability of the system.

### Metabolomics analysis by LC-MS.

The metabolomics analysis followed our previous publications with modifications (12, 22). Briefly, both RP-UHPLC–MS and HILIC-UHPLC–MS analyses were performed using an Agilent 1290 ultrahigh pressure liquid chromatography system (Waldbronn, Germany) equipped with a 6520 quadrupole-time of flight (QTOF) mass detector managed by a MassHunter workstation. For RP-UHPLC, the column used for the separation was an Agilent rapid-resolution HT Zorbax SB-C18 (2.1 by 100 mm by 1.8 mm; Agilent Technologies, Santa Clara, CA, USA). The oven temperature was set at 45°C. The gradient elution involved a mobile phase consisting of 0.1% formic acid in water (A) and 0.1% formic acid in methanol (B). The initial conditions were set at 5% phase B for 2 min with a flow rate of 0.4 ml/min. A 7-min linear gradient to 70% B was applied, followed by a 12-min gradient to 100% B, which was held for 3 min and then returned to the starting conditions over 0.1 min. The sample injection volume of 5 μl was injected, and the oven temperature was set at 40°C. For HILIC-LC, the column used for the separation was an Acquity BEH HILIC (2.1 by 100 mm by 1.7 μm; Waters Corporation, Milford, MA, USA), and the mobile phase was 10 mM ammonium acetate in 95% acetonitrile-5% water containing 0.1% formic acid (A) and 50% acetonitrile-50% water (B). The initial condition of the gradient elution was set at 100% A for 2 min, with a flow rate of 0.4 ml/min. A 13-min linear gradient to 50% B was then applied, followed by a 2-min gradient to 100% B, which was held for 3 min. The sample injection volume was 5 μl, and the oven temperature was set at 40°C.

The electrospray ionization mass spectra were acquired in positive ion mode. Mass data were collected between *m/z* 100 and 1,000 at a rate of two scans per second. The ion spray voltage was set at 4,000 V, and the heated-capillary temperature was maintained at 350°C. The drying gas and nebulizer nitrogen gas flow rates were 12.0 liters/min and 50 lb/in^2^, respectively. Two reference masses were continuously infused into the system to allow constant mass correction during the run: *m/z* 121.0509 (C_5_H_4_N_4_) and *m/z* 922.0098 (C_18_H_18_O_6_N_3_P_3_F_24_).

### Data analysis and compound identification.

Raw spectrometric data were analyzed with MassHunter qualitative analysis software (Agilent Technologies), and the molecular features, characterized by retention time, chromatographic peak intensity, and accurate mass, were obtained by using the Molecular Feature Extractor algorithm. The features were then analyzed with MassHunter Mass Profiler Professional software (Agilent Technologies). Only features with an intensity of ≥20,000 counts (approximately three times the limit of detection of our LC-MS instrument) that were found in at least 80% of the samples at the same sampling time point signal were kept for further processing. Next, a tolerance window of 0.15 min and 2 mDa was used for alignment of retention time and *m/z* values, and the data were also normalized by an internal standard. For statistical analysis, one-way analysis of variance (ANOVA) (*P* < 0.05) with Benjamini-Hochberg multiple-testing correction was employed. Fold change (FC) analysis was also performed to further filter the features, and only those features with an FC of >1.5 were selected as potential significantly altered metabolites. Unsupervised multivariate analysis PCA was performed with the Unscrambler-X statistical software package (CAMO Software, Oslo, Norway).

The structure identification of the differential metabolites was based on our published work (23, 24). Briefly, the element compositions of the metabolites were first calculated with MassHunter software from Agilent based on the exact mass, the nitrogen rule, and the isotope pattern. Then, the elemental composition and exact mass were used for open source database searching, including LIPIDMAPS (http://www.lipidmaps.org/), HMDB (http://www.hmdb.ca/), METLIN (http://metlin.scripps.edu/), and MassBank (http://www.massbank.jp/). Next, tandem MS (MS-MS) experiments were performed to obtain structural information via the interpretation of the fragmentation pattern of the metabolite. The MS-MS spectra of possible metabolite candidates in the databases were also searched and compared. Finally, the metabolites were confirmed by comparison with standards when they were commercially available. The metabolites are listed according to the minimum reporting standards for chemical analysis in metabolomics recommended by the Metabolomics Standard Initiative (MSI) (25, 26), briefly, a four-level system ranging from level 1 (identified metabolites) via levels 2 and 3 (putatively annotated compounds and compound classes) to level 4 (unidentified or unclassified metabolites that can still be differentiated based on spectrum data). For metabolic pathway analysis, metaboAnalyst (27) was used to identify relevant pathways.

## Supplementary Material

Supplemental material
