# Supplementary material for: Serum Metabolomics Investigation of Humanized Mouse Model of Dengue Virus Infection
Source: J Virol. 2017 Jun 26;91(14):e00386-17. doi: 10.1128/JVI.00386-17 (PMC5487573; doi:10.1128/JVI.00386-17)
Supplement: Supplemental material [file supp_91_14_e00386-17__index.html]

Serum Metabolomics Investigation of Humanized Mouse Model of Dengue Virus Infection — Supplemental material 

# Serum Metabolomics Investigation of Humanized Mouse Model of Dengue Virus Infection

## Supplemental material

- Supplemental file 1 -

  Fig. S1 (PCA score plots of NSG and humanized mice with dengue virus infection.)

  Fig. S2 (Pearson correlation analysis reveals correlation of reconstitution levels with two nucleosides in humanized mice.)

  Fig. S3 (Scatter plots of differential metabolites in NSG mice with dengue virus infection.)

  Fig. S4 (Pathway analysis of humanized mice with dengue virus infection using MetaboAnalyst.)

  PDF, 446K
