# Supplementary material for: Serum Metabolomics Investigation of Humanized Mouse Model of Dengue Virus Infection
Source: J Virol. 2017 Jun 26;91(14):e00386-17. doi: 10.1128/JVI.00386-17 (PMC5487573; doi:10.1128/JVI.00386-17)
Supplement: Supplemental material [file JVI.00386-17_zjv999182727s1.pdf]

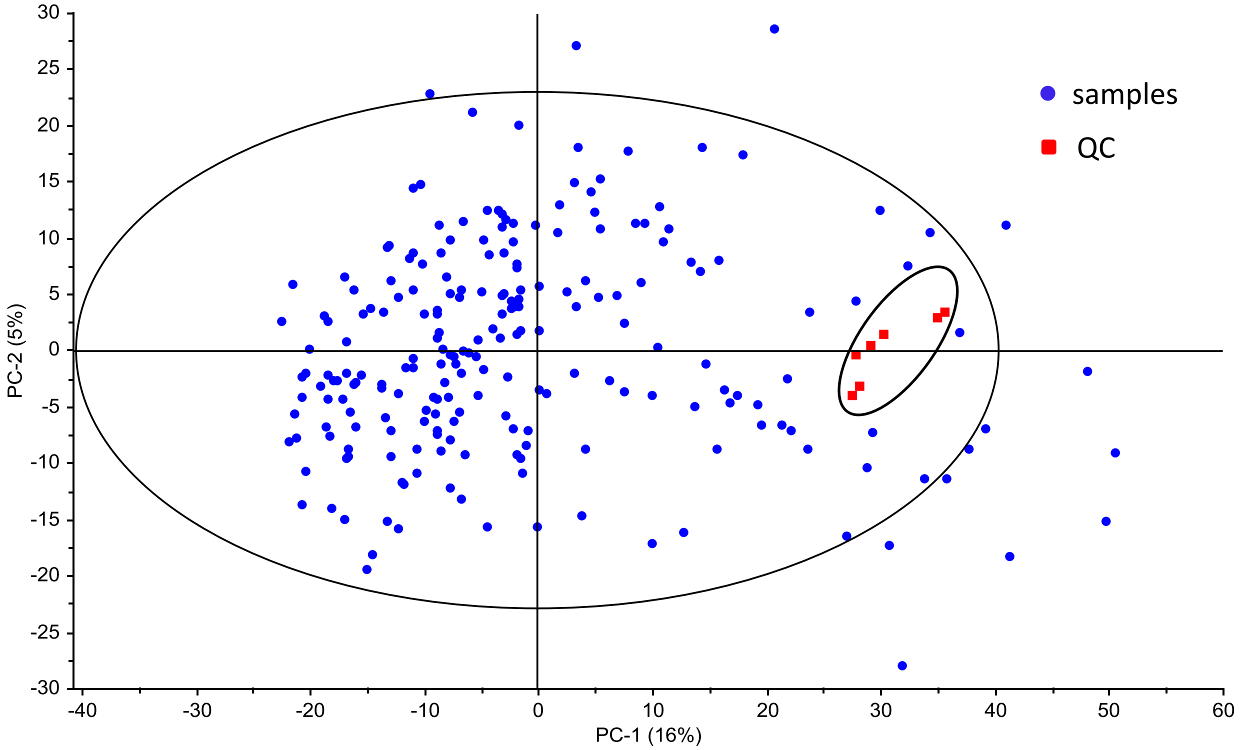

Figure S1. PCA score plots of NSG and humanized mice with dengue infection.

A

B

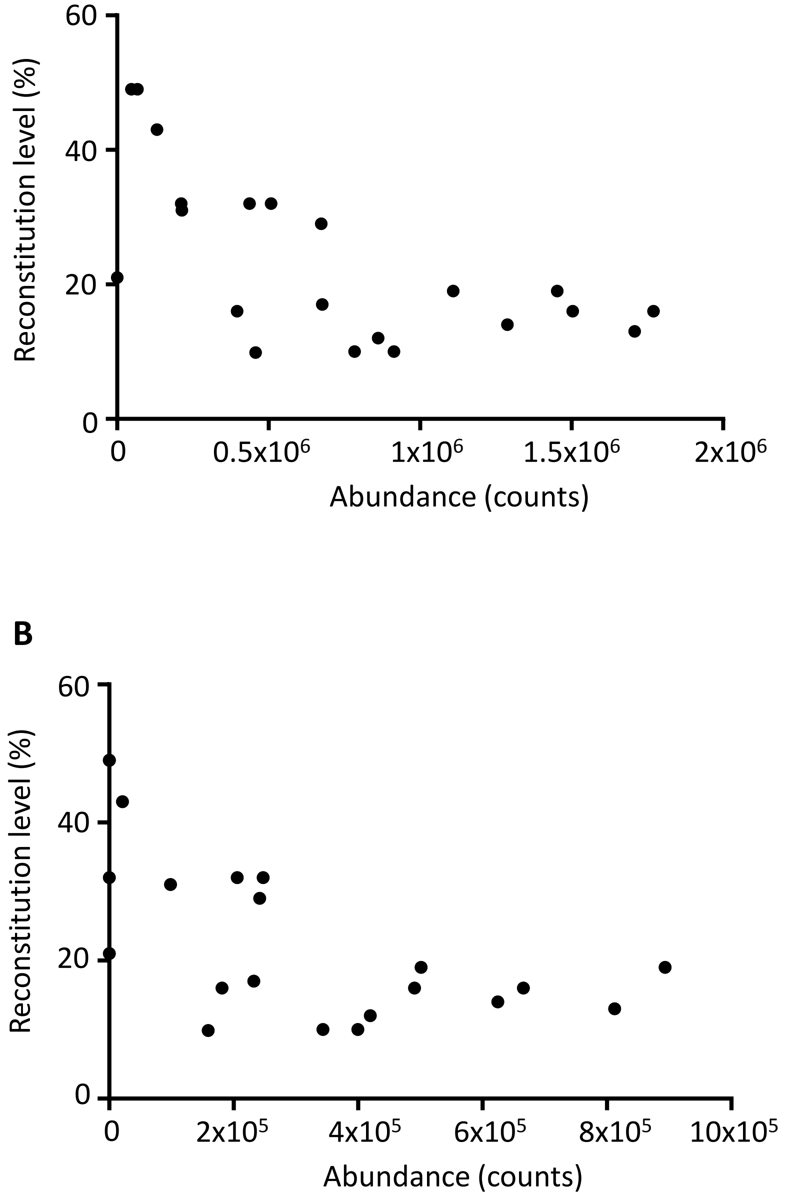

Figure S2. Pearson correlation analysis reveals correlation of reconstitution levels with two nucleosides in humanized mice. A. Negative correlation between reconstitution levels and cytidine (*r*= -0.64; *p*=0.002). **B.** Negative correlation between reconstitution levels and deoxycytidine (*r*= -0.61; *p*=0.003).

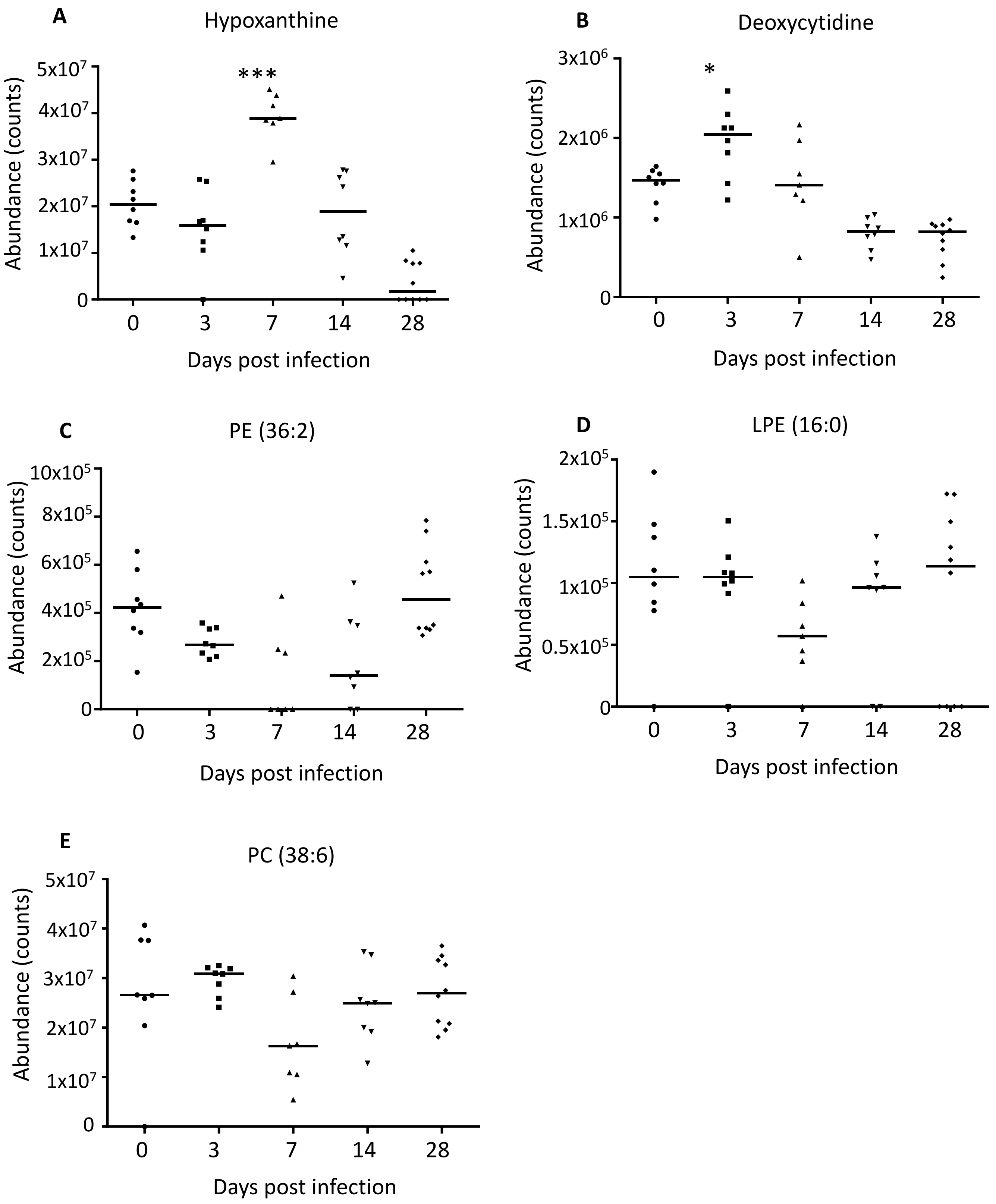

Figure S3. Scatter plots of differential metabolites in NSG mice with dengue infection. A. elevated change trend of hypoxanthine. **B.** elevated change trend of deoxycytidine. **C.** decreased change trend of PE (36:2). **D.** decreased change trend of LPE (16:0). **E.** decreased change trend of PC (38:6). * $p < 0.05$, ** $p < 0.01$, *** $p < 0.001$ by test Kruskal-Wallis test. The statistical comparison was with control levels. LPE, lysophosphatidylethanolamine; PE, phosphatidylethanolamine; PC, phosphatidylcholine.

	Total	Expected	Hits	Raw p	-log(p)	Holm adjust	FDR	Impact
Glycerophospholipid metabolism	39	0.23	3	1.27E-03	6.67E+00	1.01E-01	1.01E-01	0.23
Pyrimidine metabolism	60	0.35	3	4.41E-03	5.42E+00	3.48E-01	1.76E-01	0.03
Biotin metabolism	11	0.06	1	6.23E-02	2.78E+00	1.00E+00	1.00E+00	0.00
Aminoacyl-tRNA biosynthesis	75	0.44	2	6.85E-02	2.68E+00	1.00E+00	1.00E+00	0.06
Glycosylphosphatidylinositol(GPI)-anchor biosynthesis	14	0.08	1	7.86E-02	2.54E+00	1.00E+00	1.00E+00	0.04
Linoleic acid metabolism	15	0.09	1	8.40E-02	2.48E+00	1.00E+00	1.00E+00	0.00
Taurine and hypotaurine metabolism	20	0.12	1	1.11E-01	2.20E+00	1.00E+00	1.00E+00	0.00
Sphingolipid metabolism	25	0.15	1	1.36E-01	1.99E+00	1.00E+00	1.00E+00	0.01
alpha-Linolenic acid metabolism	29	0.17	1	1.56E-01	1.85E+00	1.00E+00	1.00E+00	0.00
Lysine biosynthesis	32	0.19	1	1.71E-01	1.76E+00	1.00E+00	1.00E+00	0.10
Phenylalanine metabolism	45	0.26	1	2.33E-01	1.46E+00	1.00E+00	1.00E+00	0.00
Primary bile acid biosynthesis	47	0.27	1	2.42E-01	1.42E+00	1.00E+00	1.00E+00	0.01
Lysine degradation	47	0.27	1	2.42E-01	1.42E+00	1.00E+00	1.00E+00	0.15
Arachidonic acid metabolism	62	0.36	1	3.07E-01	1.18E+00	1.00E+00	1.00E+00	0.00
Arginine and proline metabolism	77	0.45	1	3.66E-01	1.00E+00	1.00E+00	1.00E+00	0.10
Tryptophan metabolism	79	0.46	1	3.74E-01	9.83E-01	1.00E+00	1.00E+00	0.01
Purine metabolism	92	0.54	1	4.21E-01	8.64E-01	1.00E+00	1.00E+00	0.01

Figure S4. Pathway analysis of humanized mice with dengue infection using Metaboanalyst.
